# Discover to Deliver Pediatric Futures: Viewpoints of Future Medicine From a Pediatric Health Care System

**DOI:** 10.2196/96758

**Published:** 2026-10-20

**Authors:** Vanessa Sincock Watkins, Ken Chang, Laura Sarff, Jared Vavroch, Dawn Wolff, Lori Erickson

**Affiliations:** 1 Strategy, Innovation, and Partnerships, Children's Mercy Hospital, 2401 Gillham Road, Kansas City, MO, 64108, United States, 1 8162343000

**Keywords:** forecasting, child, strategic planning, pediatrics, future

## Abstract

Pediatric health care systems face increasing uncertainty driven by rapid technological change, workforce shifts and shortages, environmental threats, and evolving family and community needs. Pediatrics requires future-focused considerations that go beyond adult systems, including family-centered care, developmental trajectories, and long-term societal impacts on child well-being. While the future cannot be predicted, futures medicine methods offer structured approaches to anticipate change and support proactive, values-driven planning. This Viewpoint describes the application of pediatric futures methods within an academic, independent nonprofit pediatric health system and proposes a practical framework that connects future readiness to strategic decision-making and daily care delivery. Futures medicine methods are not new in pediatric health care but may not be visible from the front line of care delivery, which provides an opportunity for clinical, operational, administrative, and enterprise leaders to implement these methods in their workflows. This paper introduces the discover to deliver pediatric futures framework, a continuum that links long-range futures discovery to near-term strategic planning, operational design, implementation, and outcome demonstration, emphasizing future methods and activities for applicable use.

## Introduction

Future readiness can aid health care leaders in directional planning through numerous futures medicine methods [1]. Futures methods have varying structures, but all convene stakeholders, curate technologies, and layer future forces and capabilities, upstream strategic opportunities, and operational planning. This work is often unseen by frontline clinicians and develops alternative futures beyond 5 years from the current state. Future readiness within strategic planning for pediatric health care requires insights beyond those provided by adult health care systems’ approaches. The impact of family-centered care and pediatric-focused trends is critical in planning to create a world of well-being for all children. While we cannot predict the future, we can use futures methods to create the future we envision for all children. In a recent call to action by Mesko et al [1][Bibr R1], the authors challenged health care leaders to support the systematic application of medical futures methods across large-scale projects and dedicated expertise. In pediatric health care organizations, including our Midwest independent nonprofit pediatric health care system, futures methods are used to anticipate and prepare for future challenges and opportunities to remain competitive and resilient in an increasingly unpredictable health care environment. Pediatric care is not just applying adult models to a family-centered care model; rather, it requires an understanding of the influence of parental roles and emerging childhood conditions that may shape the future. This Viewpoint examines the application of futures medicine methods across pediatric health care and illustrates how a pediatric futures framework can guide the discovery, decision-making, design, delivery, and demonstration of optimal well-being for children within our communities and health system.

## Discover: Systematic Methods for Pediatric Futures

A systematic focus provides a basis for discovery through identifying emerging trends, signals, risks, and opportunities that may shape pediatric health and well-being across clinical, community, and societal contexts. Futures medicine methods have an established history in pediatric health care starting in 2010, supporting early discovery and long-term planning [2]. Multiple systematic methods can be used in the discovery efforts in futures medicine, including the Delphi method, scenario planning, megatrends, horizon scanning, and future forecasting. An early application of futures medicine methods came from the 2010 report by the American Academy of Pediatrics Vision of Pediatrics (VOP) 2020 Task Force [2]. The board of the American Academy of Pediatrics selected experts with a range of expertise to use the Delphi method and congregate to envision the future of pediatric health. The experts reviewed and aligned input via surveys and task force expertise, ranking upcoming trends with consolidation across the continuum of pediatric health care [2]. Scenario planning with crossing trends in a 2×2 method was used to help leadership explore the impact of potential actions in a variety of potential futures [2]. The 19 trends were consolidated into 8 critical megatrends with additional wild cards. Each megatrend had a best-case, worst-case, and most plausible scenario based on an expert consensus. Textbox 1 delineates the 8 megatrends and the current status of the futures envisioned by the VOP experts over the decades since the 2010 report. Many of the forecasted trends became reality and continue to impact us today, including pediatric workforce shortages, enhanced care delivery systems, a global pandemic, and the increasing complexity of pediatric care [3-6].

Textbox 1. **2010 forecasted megatrends from the Vision of Pediatrics 2020 Task Force.**

- Changing demographic and clinical characteristics of children and families
- Burgeoning health information technologies
- Ongoing medical advances
- Alterations in health care delivery systems
- Growth of consumer-driven health care
- Pediatric workforce dynamics
- Disasters, environmental infections, and man-made events
- Globalism

The International Society for Pediatric Innovation (iSPI) started in 2014 after the initial Delphi promulgation of the Pediatrics 2040 initiative to anticipate change in pediatric health care [7]. This global initiative continues today, providing an opportunity for pediatric stakeholders to use horizon scanning and futures thinking to anticipate the needs of the world’s healthy child [7]. The work by this coalition aligns with the STEEP (social, technological, economic, environmental, and political) framework, which encompasses technological innovations, global economics, social collaboratives, business partners, and external forces that share our interest in pediatric health impact [7]. Leadership from this collaboration has permeated globally through pediatric innovation and provides ongoing opportunities for achieving an ideal state of future pediatric health.

## Decide: Leader Decisions With Strategic Direction

The areas of discovery are applied by executive leadership and board members to determine the macro vision of an organization. The administrative leadership in an organization drives the culture of change and the evaluation of capabilities and support for the application of the discovered trends, scenarios, and vision. This practical direction decides and then supports the future focus areas of prioritization. These key strategic priorities also support plausible and preferred futures. Strategic planning is an instrument that supports leaders’ decision-making capabilities. Their expertise is enhanced from education and knowledge in strategic direction methods such as SWOT (strengths, weaknesses, opportunities, and threats) and PESTLE (political, economic, sociological, technological, legal, and environmental) [8].

The mission and vision of each organization are a key part of which prioritization areas are focused on after information has been gathered and provided by futures methods teams. While revenue streams are key in strategic decisions in health care, we also have an ethical duty to envision and create a world of well-being for our pediatric patients and their families. Traditional fee-for-service revenue models in financial planning will overlook critical innovations and future-forward programs that serve lower-income patients, including some pediatric patients [9]. The future readiness approach emphasizes combining proven care models with adaptive business models that do not rely on fee-for-service alone but are managed over time for operational and financial sustainability. Philanthropic partnerships with strategic leadership are key to success in nonprofit sustainability in health care beyond small implementation programmatic support [10].

## Design: Adaptive Strategy and Operational Alignment

As leadership shifts organizational direction to the potential futures based on the mission, culture, and financial direction of the organization, traditional design and implementation methods are used to bring the organization toward these goals [1]. Teams can intentionally create care models and technologies based on forward thinking and not only on immediate care needs or issues that have arisen in a crisis. Today, flexibility and organizational adaptability are imperative for survival, given the speed at which change is impacting the entire health care ecosystem. Leveraging STEEP factors and predictions allows health care organizations to maintain a wider aperture and focus on broad strokes with a longitudinal orientation, but it must be predicated upon our organizations’ ability to pivot and adapt on a day-to-day basis. In pediatrics, the complexity is even greater, as those who receive the care are not those who make the decision about their care [11].

For implementation and execution to be successful, strategy and planning are not enough. Strategy needs translation into operational planning and leadership from clinical leadership [8]. This includes teams knowledgeable about financial planning and analysis that can support the development of capital needs and operational and staffing support for enterprise growth. Funds flowing in strategic alignment with future readiness work combined with transparency of financial margins are a key part of health care management and sustainability where margins and reimbursement are often lower with a complex payer mix [12].

## Deliver: Dissemination and Implementation of Health Care Models

Care delivery has the biggest impact from the previous year’s planning steps, combining all the visions from future readiness at the point of operational implementation. Transparent communication of why changes are happening while supporting effective teamwork through change is a crucial role of health care organizational leadership [13]. Adapting to change while delivering care can be both complex and complicated, but our patients’ outcomes are the results, so care teams must support innovative models to drive these changes [14]. Sustained execution of innovation pilots and support for research development for areas that are not yet ready for the standard of care start with early foresight and planning for areas that will be simple, complex, or complicated as they are implemented [15]. Ongoing communication from leaders is needed to maintain complex health systems’ directional focus. Objectives and key results (OKRs) methods can be used to maintain transparent goals and commitment to leadership direction and metrics [16]. This framework supports an approach to identify, strategically measure, and operationalize objectives.

## Demonstrate: Assessing Impact and Monitoring Trends

Outcome evaluation through futures methods is continuous and requires ongoing trend monitoring. New disruptive innovations provide opportunities for a course shift toward one of the alternative futures imagined a decade prior. Ongoing benchmarking with peer children’s hospitals and real-world performance is possible with resources such as the US News Pediatric Best Hospital rankings, the Children’s Hospital Association’s annual benchmarking report, and repositories such as the Pediatric Health Information System (PHIS) database [17,18]. Ongoing outcome monitoring allows future development cycles to continue from day-to-day outcomes and helps leaders to assess their quality and outcome metrics as well as the implementation of new care models among peers [17].

## Framework Summary

The visual in Figure 1 is a collective cumulation of the process that health care leaders could use to ideate their transition to using futures medicine methods in their strategic workflow. This is a compounding framework built on a rapidly changing landscape for ideal and alternative futures that are trending in our world. The global impact and assessments of potential megatrends underlie the tough decisions that drive the administrative direction. The next megatrend is not only on the horizon but occurs simultaneously in the world and may not be exclusive to pediatric health care or the health care ecosystem. This is especially critical and more complex in a competitive environment, where pediatric providers adapt and evolve to changing landscapes, megatrends, and competitor responses over time. Ongoing outcome evaluation and demonstration of metrics are key to success in this framework. Each of the Figure 1 areas builds upon the past and is an iterative process with continual efforts and expertise along the continuum. While it is a progressive model, there is ongoing ability to flex if there are disruptive transformations that shift leadership and administrative priorities.

**Figure 1.**
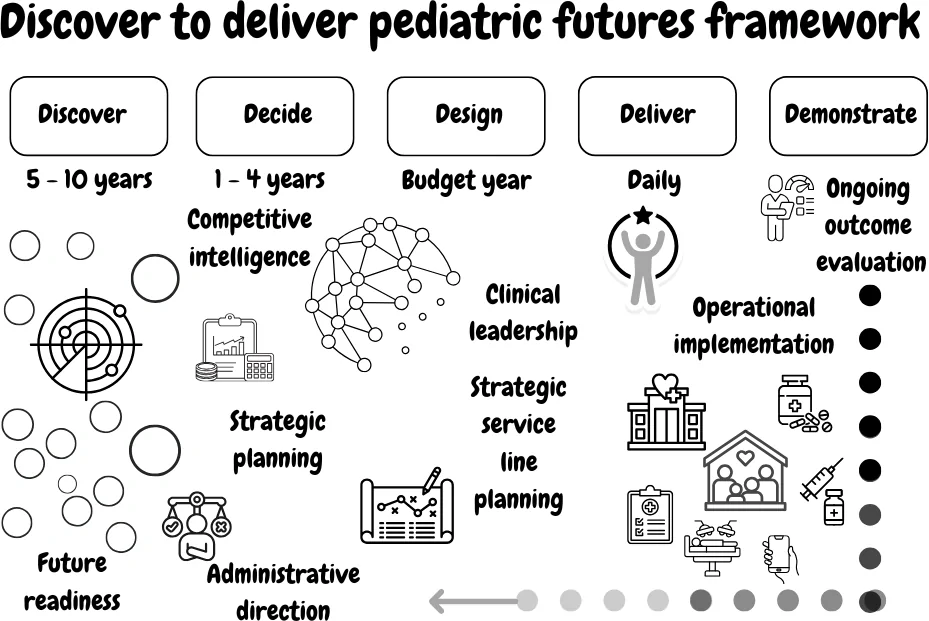
The discover to deliver pediatric futures framework. The continuum of pediatric futures methods supports the transition through potential futures and supports ongoing monitoring of trends, demonstration of outcomes, and adjustment of priorities with future methods.

## Illustrative Case Vignette: Implementation of Pediatric Futures Methods at a Pediatric Nonprofit Health Care Institution

The culture of future readiness has permeated groups such as iSPI and the VOP and created the conditions for the Strategic Services team at our institution to replicate these models within our strategy and planning approaches [2,7]. This was first implemented as our organization developed our 2020 enterprise strategic plan and, even more recently, refined as we developed our 2030 enterprise growth strategy.

### Discover

In 2018, Children’s Mercy Strategic Planning leaders began exploring the idea of introducing future readiness into their planning process, through the development of a STEEP wall to assist with innovation development, strategic planning retreats, and the upcoming development of a 2020 enterprise strategic plan. A planning analyst at the time was tasked with bringing qualitative insights that provided a wider aperture, considering significant impacts that could affect the broader pediatric health care ecosystem. The guardrails were low: think about disruptive innovation, dual transformation, human-centered design, and systems thinking. These leaders brought the first versions of future readiness to Children’s Mercy by using STEEP factors and predicted megatrends first in the development of departmental strategic planning, then our digital health strategy with diverse cross-department stakeholders, and finally 1 to 2 years later—STEEP was used as a component of informing the 2020 enterprise strategic plan. Many of these trends have been fully realized today. During this period of discovery, the chief executive officer (CEO) of 25 years retired; a new CEO began in November 2018, and we were on the cusp of the COVID-19 global pandemic.

### Decide

In 2020, the continuation of these strategies was dependent on individual staff knowledge and capacity. The organization had its first new CEO (and associated changes in senior leadership) in 25 years. As predicted by the VOP team in 2010, the COVID-19 pandemic disrupted the trajectory of many facets of life, including health care [2]. At the time, fiscal responsibility and operational excellence were key to survival; the megatrends and STEEP factors were unchanged, even after the pandemic. Some of the megatrends that remain are rapid urbanization, declining birth rates, and global warming. At the same time, as of this writing, pediatric patients’ complexity has increased, Medicaid payments have decreased, and health system consolidation has occurred both in competitive markets and across markets.

### Design

In July 2024, the principal product manager in the Center for Pediatric Innovation renewed the focus on future readiness by curating and sharing key STEEP trends, working with the strategy team on the practical application of the trends, and considering future scenarios of pediatric health care. They used the future-back method to help inform innovative projects and planning retreats. Notably, the strategic futures methods insights team was composed of interdisciplinary and cross-functional professionals coming from the fields of business, public health, bioethics, nursing, allied health, innovation, remote health solutions (remote patient monitoring), and strategy (data analytics and business planning).

### Deliver

In February 2025, the organization welcomed the current CEO following the retirement of the previous CEO [14]. Preparations to present a new future readiness concept were drafted and shared among strategy and innovation leaders (Figure 2 and Textbox 2). The organization initiated an aggressive growth plan with these discovery, decision, and process design backing theory. Additionally, our CEO brought new perspectives and considerations by way of agility, adaptability, and complexity [14]. Much of their forward-thinking leadership complements the origin story for STEEP to future readiness in the face of disruptive innovation, transformation, and human-centered design, and systems thinking.

**Figure 2.**
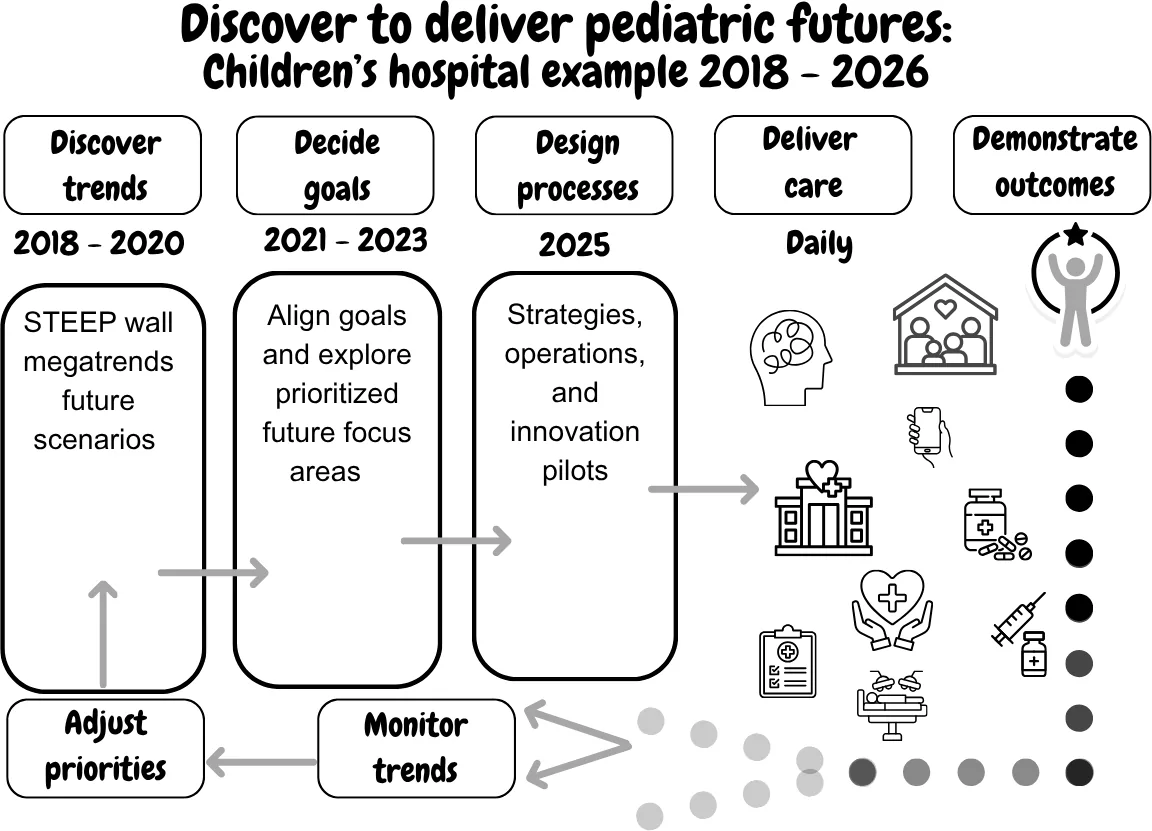
Illustrative example of the application of the framework to a nonprofit freestanding pediatric institution using pediatric futures methods. STEEP: social, technological, economic, environmental, and political.

Textbox 2. **Future readiness process.**

- Discover trends and scenarios
  - Create awareness of trends that will impact care in the next 5-10 years
  - Help translate to visualize local application and implication
  - Build future scenarios
  - Ideate the future focus area of the ideal scenario

- Decide goals
  - Align and prioritize with the following:
    - Insights and learnings
    - Capabilities and technologies
    - Competitive and market assessment
    - Assumptions
    - Initial roadmaps

- Strategy, operations, and innovation pilots
  - Strategic planning—incorporate future-back sessions for dissemination at the 5-year enterprise and departmental levels
  - Future-back workshops at the operational level with cross-departmental strategic planning sessions
  - Continued investment in innovation and research pilot programs with future-back understanding

To arrive at prioritization and strategic alignment for our enterprise strategic planning, our team began with the widest aperture possible for discovery. We examined headwinds and tailwinds impacting the broader health care ecosystem, then specifically pediatric tertiary and quaternary care, and finally particular factors impacting our system straddling 2 states. The key factors included stagnant local population growth; Medicaid uncertainty, as HR 1 (enacted July 2025) limits states’ health care–related taxes and state-directed payments used to support higher Medicaid rates for children's hospitals, putting pressure on our largest payer source and on coverage for the children and families we serve [19]; competitive threats from for-profit and private equity entities in pediatric care delivery; our aging facility infrastructure; increasing recruitment challenges for pediatric specialties and subspecialties; market consolidation; and adult health systems closing pediatric units [20]. This served as the backdrop for the “discover” trends phase and the beginning of our progression along the discover to deliver pediatric futures framework and ultimately our enterprise strategies for organizational alignment: “decide” goals.

Currently, we are beginning to take a “push” versus pull approach to democratize future readiness across the organization for all leaders. We are offering foundational learning on future readiness through future readiness deep dives, which include both short- and long-term projects; internal communications in the form of presentations; our intranet site; and a collaborative newsletter that combines competitive intelligence, future readiness, market share data, market and industry insights, and innovative communication strategies. Aligning with an executive objective, a future readiness focus was formalized by the creation of a new strategy role that would partner with innovation and teams across the enterprise. This OKR approach aligned with ongoing development of the future readiness infrastructure. Table 1 delineates the future readiness infrastructure, goals, and key aspects of implementation at our institution led by the strategic futures methods insights team. We created a STEEPLE (social, technological, economic, environmental, political, legal, and ethical) whiteboard to help inform our planning process with an emphasis on our growth plan (Figure 3).

**Table 1.** Future readiness infrastructure

Methods	Goal	Key aspects or examples
Local department education and collaboration	Monthly education forward-thinking brainstorming sessions to encourage team members to think beyond the traditional strategic, operations, physician relations, and business planning efforts	Small group setting, virtual Connect our work to our growth plan and major changes Advanced listening and reading materials are sent ahead of time and are designed to take less than 30 minutes of preparation Review team and organizational objectives and key results Strategy and forecasting podcasts, historical trend data, peer-reviewed articles, and project-specific research
Enterprise AI share group	Monthly engagement	Composed of strategy, marketing, remote health solutions, data intelligence, and other stakeholders Help AI stewards share best practices and case studies, particularly with our enterprise AI-powered chatbot and digital assistants (Microsoft Copilot)
Children’s Mercy Innovation Center Spark Bash	Yearly Innovation Center human-centered design employee fair	Employees across the enterprise submit ideas for their pediatric care innovations Submissions have a chance to gain internal support for development funding and coaching Presented a panel presentation on future technologies and trends in partnership with the innovation center, IT, and data intelligence teams Outreach table in conjunction with the Innovation Center provided information on future readiness workshop offerings
Industry and market research reports and future topics deep dives	Illustrative of the deep dives we provided to various stakeholders within strategy and by request	Examples include hospital at home, future of health care and hospitals, robotics in health care, gene therapy, bundled payments, and retail incentives Used a variety of formats tailored to the request, including the “Amazon Six Pager” format The deep dive content has been used to put forth initiatives, programs, and services for review by our Strategic Advisory Council, which includes leaders who help prioritize enterprise resources Research on the future of health care and hospitals as well as robotics in health care was directly used in the design process and deliberations of a new expansion project that is being built on the main hospital campus
Assist with retreats	Facilitate a future trends application exercise at internal planning retreats	Used trend cards to help frame health care issues and trends from other industries and to identify impacts or opportunities for our organization The trend card exercises are a fun activity to reimagine health care and access through the lens of other industries The goals for the sessions were 3-fold: get the group comfortable applying trends to projects, think broadly about how trends can impact multiple parts of the business, and internalize how each person can incorporate trends into the work they currently do Provided framework for an extension of this trend methodology for an internal director-level leadership session

**Figure 3.**
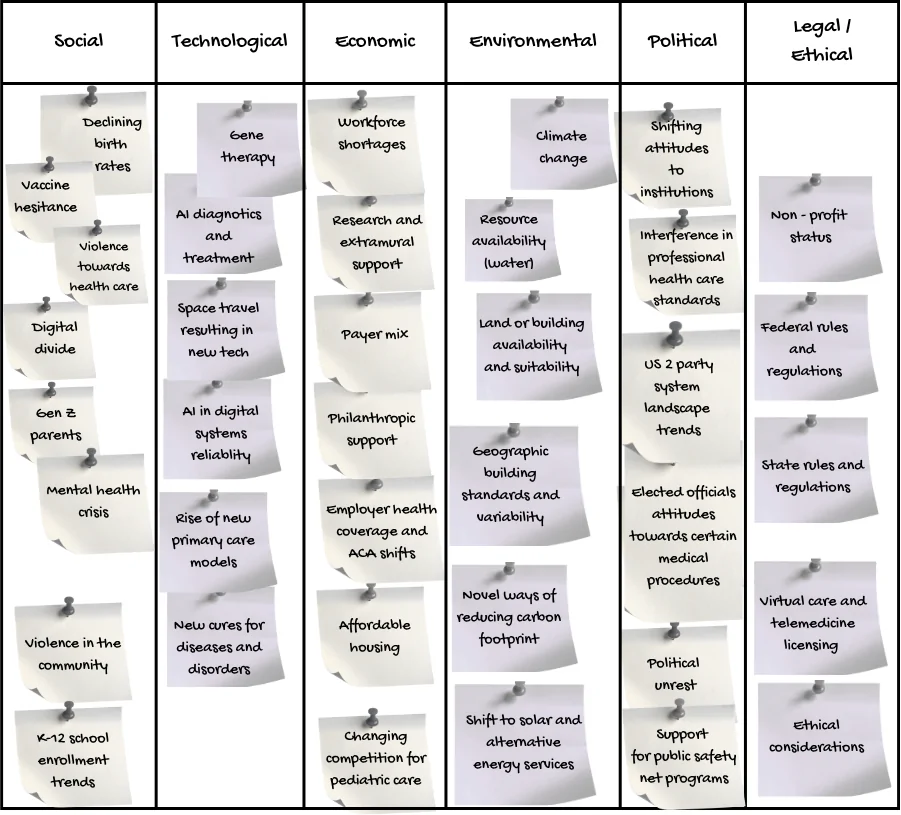
STEEPLE (social, technological, economic, environmental, political, legal, and ethical) whiteboard framework used for each goal in our growth plan.

### Demonstrate

Although the discover to deliver framework focuses on pediatric health outcomes, this illustrative case demonstrates the uptake of future methods by the enterprise team and their application to our organizational culture. We are looking ahead to the next 5 to 10 years and keeping sight of macro issues that could impact long-term planning. A key component of future readiness is continuous evaluation of current systems, care delivery, and outcomes, so priorities can be adjusted proactively rather than waiting for the next 5-year planning cycle to conclude. Health care is changing much faster than in the past, especially with the rise of information systems and AI capabilities. Ongoing demonstration of goals and metrics is required for an enterprise to be adaptable and ready for the road ahead.

This past year, we provided education and convening of future readiness concepts, looked at the growth plan through a STEEP lens, assisted with division and service line planning retreats, provided education and opportunities to connect to research, and conducted deep dives and monitoring of future trends. Through the implementation of OKRs as well as key performance indicators, we measure the utility of future readiness leading to actionable initiatives. Notably, in some cases, future readiness will not lead to a specific implementation but rather steer efforts in a general direction. As health systems move toward more agile organizations, considering the broader ecosystem within health care and outside ensures we can adapt to changes—such as new technologies (eg, AI)—that reshape how care is delivered (eg, AI identifying tumors as accurately as humans and nurse triage agent for previsit assessment), from direct patient care to research and even our future workforce [21,22].

## Health Care System Implications and Future Directions

Health care systems, especially those with pediatric care, have an ethical duty to support the future of our global population. By connecting discovery to delivery, pediatric organizations can strengthen resilience, support informed leadership decisions, and design care models and facilities that are responsive not only to current needs but also to the emerging realities that will shape child health in the years ahead. We anticipate a future state where future trends thinking and modeling are used at the individual and team levels and guided by leaders who integrate these frameworks into cultural aspects of their organization. Megatrend cards, trend slides, and STEEPLE whiteboard themes can be integrated as activities for initiative retreats and project planning. Innovative tools such as an AI-enabled chatbot and digital assistants may help staff with limited capacity or support and enable them to think more deeply with access to a greater knowledge base to further this work. The illustrative case above demonstrated feasibility and potential transferability to other health care systems with minimal infrastructure but significant future predictions. Pediatric organizations aim to collaborate on advancing digital health initiatives, such as those led by the Digital Medicine Society to create the Playbook for Pediatric Digital Medicine. Focusing on discovery and shared insights can help establish a foundation of trust while furthering the missions of each organization. This groundwork can lead to more significant collaborations that enhance health care for children [23]. While this work may be initiated by strategy teams, the use of futures methods must be embedded culturally with a democratizing approach to ideating the future of health care. Notably, these efforts could be adapted by other pediatric-focused institutions, including governmental and nongovernmental organizations at local, regional, and national levels.

## Conclusions

Applying futures methods in health care has evolved from other industries over the past decade [1]. Pediatrics is familiar with the use of future readiness, and at our institution, we have been formally undertaking these efforts for approximately 8 years, with informal involvement in global futures innovations through iSPI extending beyond that period. While the discover to deliver pediatric futures framework was drawn from a pediatric institution, other health care systems can apply this continuum to their organizations with minimal adaptation. Early education in future methods and cultural permeation of thinking beyond daily outcomes and using futures medicine methods can enhance health outcomes for all with practical, structured frameworks. Systematically engaging health organizations through their strategy and local resources, with subsequent publication of the efforts and outcomes, is essential to advancing future methods and shaping meaningful dissemination of these important contributions.

## Abbreviations

CEO: chief executive officer
iSPI: International Society for Pediatric Innovation
OKR: objectives and key result
PESTLE: political, economic, sociological, technological, legal, and environmental
PHIS: Pediatric Health Information System
STEEP: social, technological, economic, environmental, and political
STEEPLE: social, technological, economic, environmental, political, legal, and ethical
SWOT: strengths, weaknesses, opportunities, and threats
VOP: Vision of Pediatrics
